# Protein expression of CD44 in patients with meningioma tumors: association with clinicopathological parameters and survival

**DOI:** 10.1186/s43046-024-00249-9

**Published:** 2024-12-27

**Authors:** Trupti Trivedi, Neha Bhalala, Kirti Dialani, Priti Trivedi

**Affiliations:** 1 Molecular Diagnostics and Research Laboratory I, Cancer Biology Department, The Gujarat Cancer & Research Institute, Asarwa, Ahmedabad, Gujarat 380 016 India; 2 Department of Oncopathology, The Gujarat Cancer & Research Institute, Ahmedabad, Gujarat India

**Keywords:** CD44, Meningioma, Prognosis, Multivariate analysis, PFS, OS, ROC, Treatment

## Abstract

**Objective:**

Meningiomas are a molecularly ill-defined heterogeneous group of indolent intracranial tumors. Though, WHO grade 1 tumors are histologically benign, sometimes they transform into malignant and may be recurrent which remains always challenging to clinicians. Therefore, the current study sought to discover the clinical relevance of CD44 in meningioma patients.

**Method:**

Protein expression of CD44 was investigated using immunohistochemistry in a total of 70 meningioma patients. Immunoscore performed using modified H-score, CD44 expression correlated with clinicopathological parameters and progression-free survival (PFS) and overall survival (OS). Univariate and multivariate survival analysis was analyzed. The data was evaluated using SPSS statistical software and *P*-value ≤ 0.05 was considered as significant.

**Results:**

The membranous and cytoplasmic protein expression of CD44 was noted in meningioma tumors. Based on H-score, the weak (0–190 score) and strong (191–300 score) immunoreactivity was observed in 62.9% and 37.1% of patients, respectively. A statistically significant positive correlation was found between strong CD44 expression and WHO grade 2/3 tumors (*χ*^2^ = 33.551, *r* = + 0.692, *P* = 0.0001), and with the presence of brain invasion (*χ*^2^ = 19.521, *r* = + 0.528, *P* = 0.001). In Kaplan–Meier univariate survival analysis for PFS and OS, apart from WHO grade of tumors (PFS; log-rank = 12.309, *P* = 0.0001, OS; log-rank = 17.830, *P* = 0.0001) and brain invasion status (PFS; log-rank = 11.941, *P* = 0.001, OS; log-rank = 13.554, *P* = 0.0001) CD44 expression (PFS; log-rank = 14.942, *P* = 0.0001, OS; log-rank = 20.986, *P* = 0.0001) remained significant prognostic factor for PFS and OS. In multivariate survival analysis, at step 1, only CD44 remained independent prognosticator for PFS (HR = 11.014, 95% CI = 2.256–23.602, *P* = 0.001) and OS (HR = 8.553, 95% CI = 2.831–25.847, *P* = 0.0001). In relation to treatment offered, patients with strong CD44 expression and if treated with surgery followed by adjuvant radiotherapy showed a high incidence of death (log-rank = 13.402, *P* = 0.0001) as compared to patients treated with surgery only. Receiver operating characteristic (ROC) curves also confirmed a good efficacy of CD44 as a prognosticator for disease outcome (PFS; *P* = 0.0001, OS; *P* = 0001).

**Conclusion:**

Our overall findings addressed that a study of CD44 protein expression would be beneficiated to meningioma patients from unnecessary overtreatment and drug-induced toxicity. Also, CD44 could be one of the promising biomarkers that might differentiate high-risk meningioma patients for better treatment management.

## Background

Meningiomas are a molecularly ill-defined heterogeneous group of indolent tumors, accounting for 37.1% of all primary intracranial tumors. These tumors are classified into three grades based on the World Health Organization (WHO). Approximately 80% of these tumors are grade I (benign), while the remaining 20% are either grade II (atypical) or grade III (malignant) tumors. These indolent tumors are mostly benign in nature, though, they have a tendency to recur or even sometimes transform into more malignant tumors-atypical or anaplastic with brain invasion [1]. Accordingly, grade II and grade III tumors are considered more aggressive in nature, and they have poor prognosis. The most reliable clinical variables are the histopathological WHO grade of tumors and the extent of resection at the time of surgery, 7–25% of grade I tumors have been correlated with recurrence [2]. Demonstrating these parameters does not always correlate with clinical aggressiveness and may sometimes fail to accurately predict the clinical behavior and long-term recurrence. Thus, the accuracy of predicting recurrence remains a considerable challenge [3].

Meningiomas are molecularly ill-defined tumors probably because of the lack of reported studies on the molecular profiling of these tumors [2]. Recently, many studies have focused on biological mechanisms that may explain different tumor behaviors and serve as prognostic biomarkers or even could be a target in therapeutic strategy. However, still there is lacking clinically amenable potential prognostic biomarkers that can predict early recurrence, response to therapy and identify high-risk patients for clinical management.

CD44, a transmembrane cell surface glycoprotein, is normally expressed in all human cells. It is primarily involved in regulating cell adhesion, migration, angiogenesis, proliferation, and inflammation [4]. It may take part in the process of tumorigenesis as CD44 and its variants have a potential role in the development of malignant tumors [5]. However, there is a debate regarding its role in carcinogenesis, as it has a role in promoting the pro-tumorigenic signaling pathways and advancing the metastatic cascade. On the other hand, its role has been reported to suppress growth and metastasis. Initially, CD44 was defined for hematopoietic stem cells, it has since been confirmed as a marker of cancer stem cells [6]. Infect, CD44 is the first identified cancer stem cell marker accounting for tumor progression in multiple cancers [5]. In recent years, CD44 has gained significant attention because of its usefulness as a stem cell marker and has surfaced as a budding therapeutic target, imposing a greater understanding of CD44 in various cancers [7]. However, in meningiomas, a paucity of studies have investigated CD44 expression, and that too, mainly focused on the relationship between CD44 and proliferative ability [8]. Thus, the correlations between CD44 protein expression and the clinicopathological parameters and disease outcome of meningiomas have not been fully investigated. Hence, the present study sought to investigate the protein expression of CD44 using immunohistochemistry in meningioma tumors to correlate the expression of CD44 with clinicopathological parameters, to identify its relation with treatment offered to the meningioma patients, and to explore its impacts on disease outcome.

## Methods

### Patient selection and follow-up details

A total of 70 untreated histologically confirmed meningioma patients with WHO grade 1-3 tumors registered at our Institute from January 2013 to January 2018 were enrolled in the current study. The formalin-fixed paraffin-embedded tissue blocks (FFPE) of meningioma patients archived from the Oncopathology Department of our Institute, The Gujarat Cancer & Research Institute, Ahmedabad, India. The study was approved by the Institute’s Ethics Committee, Institutional Review Board (IRB), and Institutional Review Committee (IRC). (Approval No: IRC/2024/P-62). The written consent forms were obtained from all the enrolled patients. Detailed clinical and pathological history of the patients was obtained from the case files maintained at the Medical Record Department of our institute. The clinicopathological characteristics of the enrolled patients are listed in Table 1.

**Table 1:** Clinical characteristics of meningioma patients

Characteristics	*N*	%
Total patients	70	
Age (median 50 years, range 19–70 years)		
≤ 50	41	58.6
> 50	29	41.4
Gender		
Female	46	65.7
Male	24	34.3
Tumor location		
Frontal	35	50.0
Occipital	10	14.3
Parietal	04	5.7
Temporal	03	4.3
Others	18	25.7
WHO grade of tumors		
Grade 1	50	71.4
Meningothelial	29	58.0
Fibroblastic	09	18.0
Transitional	08	16.0
Angiomatous	03	6.0
Psammomatous	01	2.0
Grade 2	19	27.1
Clear cell	10	52.6
Atypical	08	42.1
Chordoid	01	5.3
Grade 3	01	1.4
Anaplastic	01	100
Brain metastasis invasion		
Absent	51	72.9
Present	19	27.1
Treatment		
Only surgery	52	74.3
Followed by radiotherapy	18	25.7
Recurrence		
No	56	80
Yes	14	20
Survival		
Alive	51	72.9
Dead	19	27.1
CD44 expression-H-Score (median 190)		
Weak (0–190 score)	44	62.9
Strong (191–300 score)	26	37.1

In the present study, more than 50% of patients were ≤ 50 years (range 19–70 years). The majority of patients were females, with a female: male ratio of 2:1. Fifty percent of patients had tumors at the frontal lobe of the brain and 71% (50/70) patients had grade I tumors. Twenty-seven percent and 1.4% (1/70) patients had grade 2 and 3 tumors, respectively. As in the grade 3 group, only one patient was enrolled and also, grade 2 and 3 tumors are in the malignant group category, for data analysis, we clubbed grade 2 (*n* = 19) and grade 3 (*n* = 01) tumors. Based on histological subtypes, 29 meningothelial (58%), 9 fibroblastic (18%), 8 transitional (16%), 3 angiomatous (6%), and 1 psammomatous (2%) variants were observed in grade 1 tumors. In grade 2 tumors, 10 clear cells (52.6%), 8 atypical (42.1%), and 1 chordoid (5.3%). One patient with a grade III tumor showed an anaplastic type of tumor (Table 1). Also, based on more than 70% of patients showed absence of brain invasion. Primary treatment offered to the patients was surgery (100%, 70/70), and surgery followed by radiotherapy as adjuvant therapy was undertaken by 25.7% of patients. Surgical procedures performed by the Neuro-oncology Department of this Institute.

Survival analysis for progression-free survival (PFS) and overall survival (OS) was evaluated for a minimum of 48 months and patients who could be followed for a minimum period of 48 months or until their death within that period were included. Within 48 months, 20% (14/70) patients had developed recurrent disease, and 27% (19/70) patients died within that period (Table 1).

### Immunohistochemistry

CD44 protein expression was studied using immunohistochemistry (IHC) described previously in our earlier study [9]. Formalin-fixed paraffin-embedded tissue blocks retrieved from the tissue repository of our institute’s Onco Pathology Department. The blocks were cut into 4 μm sections and mounted on 3-amino propyl triethoxy silane (APES)-coated slides. The staining was performed using an HRP/DAB (ABC) Detection IHC kit (Abcam, Cambridge, UK) according to manufacturer protocol. Briefly, antigen retrieval treatment was given by heating the sections in 10 mM sodium citrate buffer (pH 6.0) in a pressure cooker. Then, sections were incubated overnight at 4 °C with the primary mouse monoclonal antibody of anti-CD44 (HCAM-P2A1:sc53298) from Santa Cruz biotechnology with 1:100 dilution in TBS. The stained sections were mounted with DPX and observed under the light microscope. Sections with intense staining for CD44 were used as positive control, whereas negative control was obtained by omission of primary antibody. Membranous and cytoplasmic staining was observed for meningioma tumor cells.

The scoring system has already been published in our previous literature [9]. All sections were scored independently by two independent researchers in a blinded fashion. The staining intensities and the percentage of positive cells for staining were separately assessed in primary tumor tissues. The CD44 H-score was counted by multiplying the intensity level by the percentage of positive cells resulting in a value between 0 and 300. Accordingly, the median *H*-score value for the CD44 protein expression was 190 for meningioma tissue specimens. Data were divided into weak (0–190 score) and strong (191–300 score) CD44 protein expression groups by median *H*-score value.

### Statistical analysis

Statistical analysis was carried out using SPSS statistical software version 27 (SPSS Inc., USA). Two-tailed *χ*2 test was used to assess the association between the two parameters. The correlation between two parameters was calculated using Spearman’s correlation coefficient (*r*). In the case of a patient number less than 5 in the cells of 2 × 2 tables, Yates’ Continuity correction value along with its significance was taken into consideration. The receiver’s operating characteristic (ROC) curve was constructed to determine the discriminating efficiency of CD44 between high and low-risk patients for recurrence and disease outcome. Univariate survival analysis was carried out by the Kaplan–Meier method and log-rank statistics were used to assess the prognostic significance of PFS and OS. Multivariate survival analysis was performed using the Cox forward step-wise regression model. The Wald statistic and hazard ratio (HR) with a 95% confidence interval (CI) were used to assess risk for overall survival. *P*-values ≤ 0.05 were considered significant.

## Results

### CD44 expression and its association with clinicopathological parameters

**Fig. 1:**
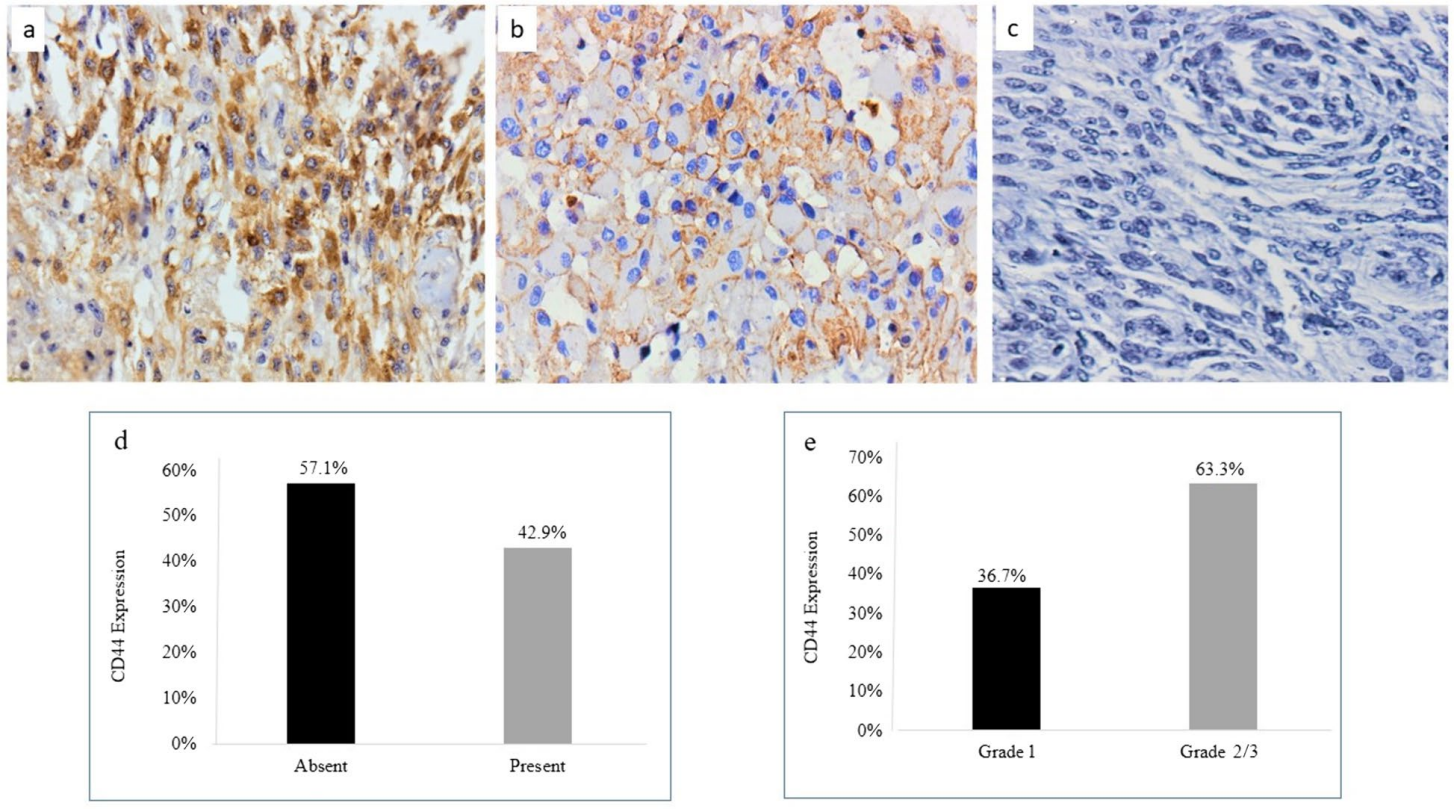
Incidence of CD44 in meningioma tumors. **a** Representative image of cytoplasmic staining of CD44 in meningioma tumors. **b** Representative image of membranous staining of CD44 in meningioma tumors. **c** Representative image of negative staining of CD44 in meningioma tumors. **d** Incidence of CD44 in meningioma tumors. **e** CD44 protein expression in WHO Grade 1 and Grade 2/3 meningioma tumors

The membranous and cytoplasmic staining was observed for CD44 protein expression (Fig. 1) in meningioma tissues. Based on the H-score, weak and strong CD44 immunoreactivity was noted in 62.9% (44/70) and 37.1% (26/70) of patients. Two-tailed chi-square test was used to evaluate the association of CD44 protein expression with clinicopathological characteristics. We found that a high incidence of strong CD44 protein expression was significantly associated with the grade of tumors and brain invasion status. Ninety percent of meningioma patients with grade 2/3 tumors showed significantly higher expression of strong CD44 expression compared to 16% of patients with grade I tumors (*χ*2 = 33.551, *r* = + 0.692, *P* = 0.0001) (Table 2). Also, 78.9% of patients who had an incidence of brain invasion showed strong expression of CD44 compared to patients with an absence of brain invasion (*χ*2 = 19.521, *r* = + 0.528 *P* = 0.001) (Table 2).

**Table 2:** Association between CD44 immunoreactivity with clinicopathological parameters of meningioma patients

CD44 expression in meningioma tumors (Median-190)						
Variables	*N*	Weak *N* (%)	Strong *N* (%)	*χ*2	*r*	*P*-value
Age (years)						
≤ 50	41	26 (63.4)	15 (36.6)	0.013	0.014	0.910
> 50	29	18 (62.1)	11 (37.9)			
Gender						
Male	24	13 (54.2)	11 (45.8)	1.181	0.130	0.284
Female	46	31 (67.4)	15 (32.6)			
Location of tumors						
Frontal	35	23 (65.7)	12 (34.3)	4.528	000	1.000
Occipital	10	04 (40)	06 (60)			
Parietal	04	02 (50)	02 (50)			
Temporal	03	03 (100)	00 (00)			
Others	18	12 (66.6)	06 (33.3)			
WHO grade of tumors						
Grade 1	50	42 (84)	08 (16)	33.551	0.692	**0.0001**
Grade 2/3	20	02 (10)	18 (90)			
Brain invasion						
Absent	51	40 (78.4)	11 (21.6)	19.521	0.528	**0.001**
Present	19	04 (21.1)	15 (78.9)			

### Survival analysis

Survival analysis for PFS and OS was performed including all clinicopathological parameters and CD44 protein expression. Univariate and multivariate survival analysis was carried out using the Kaplan–Meier survival function and Cox regression forward stepwise model, respectively.

### Univariate survival analysis for PFS and OS using Kaplan–Meier survival analysis

#### Progression-free survival

**Fig. 2:**
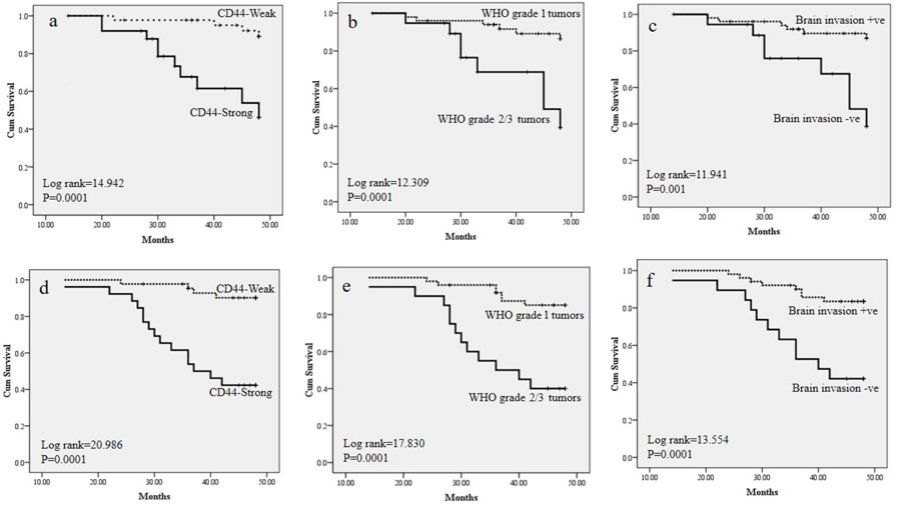
Kaplan–Meier survival curves for progression-free survival and overall survival for meningioma patients. **a** Patients with strong expression of CD44 had significantly reduced PFS. **b** Patients with WHO grade 2/3 tumors showed a higher incidence of relapse than patients with grade 1 meningioma tumors. **c** Patients with the presence of brain invasion had a significantly high incidence of recurrence within 48 months. **d** Patients with strong expression of CD44 had significantly poorer OS than patients with weak CD44 expression. **e** Patients with grade 2/3 WHO grade tumors showed a higher incidence of death rate than patients with grade I meningioma tumors. **f** Patients with the presence of brain invasion had a significantly higher incidence of death as compared to patients with the absence of brain invasion

The Kaplan–Meier survival system for PFS demonstrated that 40% of patients with grade 2/3 tumors (log-rank = 12.309, df = 1, *P* = 0.0001) and 42% of patients with the presence of brain invasion (log-rank = 11.941, df = 1, *P* = 0.001) showed reduced PFS compared to their respective counterparts (Table 3). Also, the survival curve for PFS indicated that 38% (10/26) of patients with strong CD44 expression had significantly reduced PFS compared to 9% of patients with weak CD44 expression (log-rank = 14.942, df = 1, *P* = 0.0001) Fig. 2a; Table 3). A similar significant difference in PFS we noted for WHO grade of tumors and brain invasion status when analyzed using Kaplan–Meier survival curves (Fig. 2b, c).

**Table 3:** Univariate survival analysis for PFS and OS using Kaplan–Meier analysis

		Progression-free survival			Overall survival		
Parameters	*N*	Disease relapsed	Log-rank	*P*-value	Patients died	Log-rank	*P*-value
Age		N (%)			*N* (%)		
≤ 50	41	09 (21.9)	0.215	0.942	11 (26.8)	0.009	0.924
> 50	29	05 (17.2)			08 (27.5)		
Gender							
Male	24	10 (41.6)	0.369	0.544	06 (25)	0.065	0.799
Female	46	04 (8.6)			13 (28)		
Location of tumors							
Frontal	35	07 (20)	1.263	0.868	08 (25)	2.939	0.568
Occipital	10	02 (20)			04 (40)		
Parietal	04	00 (00)			02 (50)		
Temporal	03	01 (33.3)			00 (00)		
Others	18	04 (22.2)			05 (27.7)		
WHO grade of tumors							
Grade 1	50	06 (12)	12.309	**0.0001**	07 (14)	17.830	**0.0001**
Grade 2/3	20	08 (40)			12 (60)		
Brain invasion							
Absent	51	06 (11.7)	11.941	**0.001**	08 (15.6)	13.554	**0.0001**
Present	19	08 (42.1)			11 (57.8)		
CD44 expression							
Weak	44	04 (09)	14.942	**0.0001**	04 (09)	20.986	**0.0001**
Strong	26	10 (38.46)			15 (57.6)

#### Overall survival

The Kaplan–Meier curve for OS demonstrated that a significantly high incidence of death was noted in patients with grade 2/3 tumors (log-rank = 17.830, df = 1, *P* = 0.0001) and the presence of brain invasion (log rank = 13.554, *P* = 0.0001), than patients with their respective counterparts (Table 3). Also, the survival curve for OS indicated that 57.6% (15/26) of patients with strong CD44 expression had significantly reduced PFS compared to 9% of patients with weak membranous CD44 expression (log-rank = 20.986, df = 1, *P* = 0.0001) Fig. 2d; Table 3). A similar significant difference we noted for OS in grade of tumors and brain invasion status when analyzed using Kaplan–Meier survival curves (Fig. 2e, f).

### Multivariate survival analysis

#### Progression-free survival

Multivariate survival analysis using the Cox-forward stepwise proportional hazard regression model for PFS indicated that CD44 protein expression entered at step 1 (HR = 11.014, 95% CI = 2.256–23.602, *P* = 0.001). Thus, only CD44 remained an independent risk predictor for meningioma patients (Table 4).

**Table 4:** Multivariate survival analysis using Cox-forward step-wise regression model for PFS and OS

Survival	Step	Parameter	HR	Wald	95% CI		*P*-value
					Lower	Upper	
PFS	1	CD44	11.014	7.297	2.256	23.602	**0.001**
OS	1	CD44	8.553	4.470	2.831	25.847	**0.0001**

#### Overall survival

Multivariate survival analysis for OS showed that CD44 protein expression entered at step 1 (HR = 8.553, 95% CI = 2.831–25.847, *P* = 0.0001). Thus, only CD44 remained an independent prognosticator that could predict shorter OS for meningioma patients (Table 4).

#### Impact of CD44 on disease status in relation to treatment

Kaplan–Meier univariate survival analysis for PFS revealed a significantly reduced relapse rate in patients with strong CD44 protein expression when treated with surgery only rather than surgery followed by adjuvant therapy. However, this association marginally failed to reach statistically significant (log-rank = 3.625, *P* = 0.057) (Table 5).

**Table 5:** Survival analysis in relation to treatment offered

		Progression-free survival		Overall survival	
Parameter	*N*	Disease relapsed	Log-rank *P*-value	Patients died	Log-rank *P*-value
		N (%)		N (%)	
CD44 (S)	**52**	04 (7.6)	3.625 0.057	10 (19.2)	13.402 **0.0001**
Weak	39	01 (2.5)		03 (7.6)	
Strong	13	03 (2.3)		07 (53.8)	
CD44 (S + RT)	**18**	10 (55.5)		09 (50)	
Weak	05	03 (60)		01 (20)	
Strong	13	07 (53.8)		08 (61.5)	

*S* surgery, *RT* radiotherapy

In relation to OS, Kaplan–Meier survival analysis showed that patients with strong CD44 protein expression had a significantly inferior death rate when treated with surgery followed by adjuvant therapy than patients treated with surgery only (log-rank = 13.402, *P* = 0.0001) (Table 5).

### Receiver operative characteristic analysis (ROC) for CD44 expression in meningioma patients

**Fig. 3:**
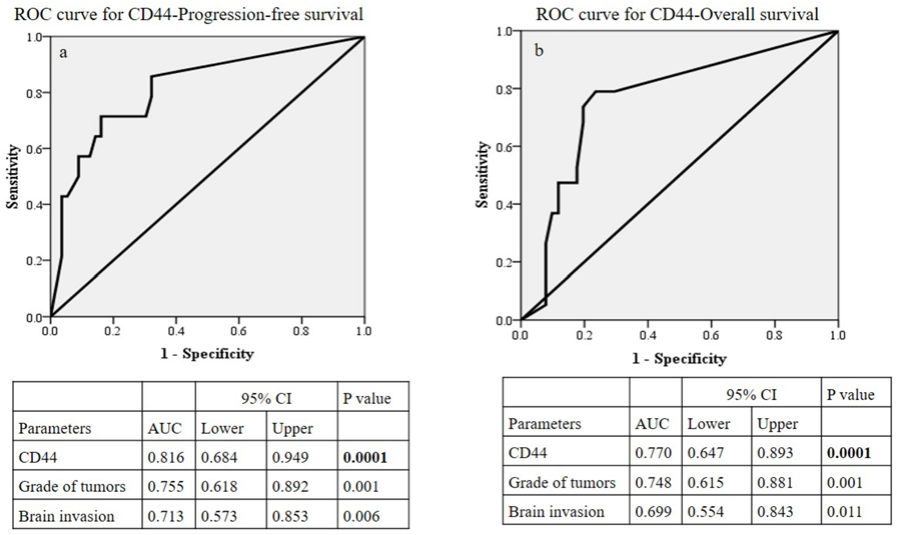
ROC curves for CD44 discriminating high-risk patients for recurrence and death. **a** ROC curve for CD44 showing efficacy to discriminate high-risk patients for recurrence. **b** ROC curve for CD44 showing efficacy in discriminating high-risk patients for death

ROC curve analysis was constructed to determine the discriminating efficiency of CD44 between high and low-risk patients for recurrence and disease outcome. The ROC curve also confirmed that CD44 showed a strong discriminatory efficacy between high and low-risk patients for disease recurrence (AUC = 0.816, 95% CI = 0.684–0.949, *P* = 0.0001) and overall survival (AUC = 0.770, 95% CI = 0.647–0.893, *P* = 0.0001, Fig. 3a, b).

## Discussion

CD44, a cell surface marker, is structurally integrated with merlin protein in the cell membrane of meningioma tumors, and its overexpression may be associated with the invasiveness and anaplasia of meningioma. It is a marker that could be used to facilitate histological detection and assist in brain brain-invasive growth of meningioma. However, there is a dearth of data available that shows the clinical impact of CD44 in meningioma. Therefore, the present study sought to explore the clinical impact of CD44 on meningioma patients.

We examined the correlation between CD44 protein expression with clinicopathological variables and the survival of patients. In addition, we determined the impact of CD44 in relation to treatment offered to patients with disease outcomes. Using ROC curves validated the efficacy of CD44 that discriminates the high and low-risk patients that develop recurrence and death during 48 months. Using immunohistochemistry the protein expression of CD44 was investigated. Our study illustrated CD44 as a significant independent marker for meningioma patients that could be able to identify high-risk meningioma patients. Strong expression of CD44 showed a positive association with WHO high-grade tumors and the presence of brain invasion. Also, in multivariate survival analysis, we showed that only CD44 remained the most potentially significant prognostic factor to predict reduced PFS and shorter OS. As per our knowledge, for the first time, our study showed the impact of CD44 in relation to treatment offered to meningioma patients, where we found that patients with strong CD44 protein expression had a significantly lower incidence of recurrence and death rate if they were treated with surgery only than if patients treated with surgery followed by adjuvant therapy. Thus, CD44 might be helpful to prevent patients from unnecessary overtreatment and thereby prevent them from drug-induced toxicity.

In the existing study, the majority of patients were female in gender, and that was higher than males, with female: male ratio found was 2:1. Out of 70 patients, 65.7% of patients were females. Meningioma is the only cerebral tumor whose predominance is female with a sex ratio estimated at 2:1 [10]. This figure was similar to that reported by Mostfa and Khairy [11] and Gassoum et al. [12] where females constituted 61.9% of their patients with a female: male ratio of 1.6:1. The association between hormone receptor expression and meningiomas has been used to explain the discordant prevalence of meningiomas in females, where the overall ratio is 2:1 in the brain [13, 14].

There has long been an association between hormone receptors expressed on meningiomas and their increased frequency among female patients, although the data has been highly variable. Pregnancy may be responsible for the sudden clinical onset of intracranial meningiomas because of the hormone-related tumor changes. Contraceptive and fertilization therapies, mainly with progesterone, should be avoided in patients with known meningiomas because of the risk of symptom occurrence and tumor progression [15]. On the contrary, Korhonen et al. [16] reported the higher incidence of meningiomas in women cannot be explained by differences in sex hormone receptors. In one meta-analysis, Benson et al. [17], reported significantly increased risk for all CNS tumors, including glioma and meningioma in users of estrogen-only as hormonal therapy. However, women taking estrogen-progestin therapy showed no increase in risk. Recently, Agopiantz et al. [18] have shown that progesterone receptors are currently the strongest parameter that has a role in meningioma development in females, however, the data on estrogen and androgen receptors are still patchy and require further study. Thus, the high incidence of meningioma in women cannot be explained only by differences in sex hormone receptors and thus other hidden causes should be looked for.

CD44 is a transmembrane protein, so the delocalization of CD44 to the cytoplasm may cause alterations in the cell–cell and cell-extracellular matrix interactions. Accordingly, cytoplasmic staining in this series may reflect the production of aberrant CD44 proteins by the malignant cells [19]. Various previous data indicated some differences in biological function and timing of expression of membranous and cytoplasmic CD44. In the present study, we also observed membranous and cytoplasmic staining of CD44, however, cytoplasmic staining was focal and was noted in a few cases of grade 1 tumors. Interestingly, the grading of meningiomas depends mainly on the invasion of adjacent structures. Therefore, extensive CD44 membranous expression in high-grade meningioma may reflect a tendency toward more invasive power of neoplastic cells into surrounding structures. These findings strengthen the putative role of membranous CD44 protein in the cellular progression of meningiomas.

Based on the modified H-score, the protein expression of CD44 was categorized into weak (0–190 score) and strong (191–300 score) categories and data was correlated for significance. Accordingly, weak and strong expression of CD44 was noted in 62.9% and 37.1% of meningioma patients. When correlated with clinicopathological parameters, a significantly positive correlation was found between high-grade tumors (grade 2/3 tumors) and the presence of brain invasion status. A strong expression was observed in grade 2/3 tumors. Ninety percent of patients with grade 2/3 tumors showed strong expression of CD44. However, in grade 1 tumors it was observed in 16% of tumors (*P* = 0.0001). The expression of CD44 in meningioma patients with intracranial meningiomas is still controversial. Few studies demonstrated high expression in high-grade meningiomas [11, 20], and another contradictory report showed that the expression is more extensive in benign meningioma than atypical meningioma [21, 22] Concordance to our results, the significant difference in CD44 expression was reported by Mostafa et al. [11] with 81.8% positivity in grade 2 tumors and 18.2% in grade 1 tumors. These results coincide with a study done by Trenda et al. [23] and Arsene et al. [22], where a significant parallel increase in CD44 expression in high-grade meningioma tumors compared to benign meningioma. Interestingly, some studies reported a decline in CD44 expression in high-grade meningiomas Figarella-Branger et al. [21] and Arsene et al. [22]. Freitag et al. [24] reported a significantly higher expression of CD44 mRNA in low-grade tumors than in high-grade meningiomas. This discrepancy between CD44 results in the literature may be reasoned to several possibilities. Importantly, the heterogeneity of CD44 proteins in meningiomas is secondary to its post-translational modifications and differential splicing [25]. Also, the variability in the scoring methodologies among the different studies explains this discrepancy. Extensive CD44 expression in high-grade meningioma may reflect a tendency toward more invasive power of meningioma cells into surrounding structures [26]. CD44 functions in supporting tumor progression and aggressiveness can be attributed to its diverse binding ligands. In our study, strong CD44 expression correlated positively with high-grade meningioma tumors thus CD44 is a marker of aggressiveness as it is expressed in high grades and plays a major role in tumor progression.

Meningiomas are mostly benign brain tumors; however, they have a tendency to recur or even sometimes transform into more malignant tumors-typical or anaplastic with brain invasion. In this study, we observed a positive correlation between CD44 and the presence of brain invasion status. Brain invasion is defined as irregular projections of meningioma into adjacent brain tissue without an intervening layer of leptomeninges, and can be assessed only by histopathologic examination of the meningioma surgical specimen. Many lines of evidence indicate that the interaction between CD44 and hyaluronic acid-mediated tumor invasiveness and migration in various cancers [27, 28]. In a study by Pizem et al. [29], brain invasion was reported in 42.3% of meningioma patients, only when there were irregular projections of meningioma into adjacent brain tissue without an intervening layer of leptomeninges regardless of its extent. In the latest WHO CNS5 classification [30], brain-invasive benign meningioma is considered grade 2, and the study by Perry et al. [31] reported evidence that brain-invasive meningioma has a similar prognosis to atypical meningioma. In concordance with our results, a positive correlation between CD44 and brain invasion was noted by Mostafa et al. [11] and Figarella-Branger et al. [21] without statistically significant results mentioned in their studies.

The invasive activity is based on the interaction of the extracellular domain of CD44 with the extracellular matrix. CD44 can act as an intracellular signaling molecule by enhancing the expression of CD44 intracellular domain to maintain and increase the stemness of these stem-like cells. Many studies have demonstrated an important role for hyaluronan-CD44 in epithelial-mesenchymal transition (EMT). One of the important characteristics of EMT is in ability to invade and metastasize. The grading of meningioma depends mainly on the invasion of adjacent structures of the brain. Taken together extensive CD44 membranous expression in high-grade meningioma may reflect a tendency towards more invasive power of neoplastic cells of surrounding structures. These findings strengthen the putative role of membranous CD44 protein in the cellular progression of meningioma.

Next, we investigated the potential role of CD44 as a prognostic marker in meningiomas. Out of a total of 70 meningioma patients, 20% (14/70) patients developed recurrent disease and 27% (19/70) patients died within that period (Table 1). Most interestingly, out of 14 patients who developed recurrence, 6 patients (42.9%) had grade 1 tumors and out of 6 grade 1 patients, 50% (3/6) patients showed strong CD44 immunoreactivity. Strong CD44 expression showed reduced PFS and inferior OS. A study by Abd Elhakeem et al. [26] reported that CD44 expression was a poor prognostic factor for meningioma patients. In contrast, Kamamoto et al. [20] reported the lack of correlation between CD44 protein expression and OS, however found that high expression of CD44 shows a tendency towards shorter PFS (*P* = 0.0563). Jijiwa et al. [32] reported that expression of CD44 has been indicated to be correlated with poor survival. CD44 is significantly associated with poorer prognosis in many cancers including breast, gastric, ovarian, and oral cancers. In a study by Boxberg et al. [33], they reported CD44 as an independent prognostic factor for poor OS and PFS in patients with advanced oral cancer. In a study by Lin et al. [34] they revealed that CD44 expression was significantly associated with high TNM stage and poor OS in ovarian cancer. Zanjani et al. [35] also reported that high CD44 overexpression is statistically associated with more aggressive tumor behavior, tumor grade, and poor survival in clear renal cell carcinoma. We also evaluated the impact of CD44 in relation to treatment offered to meningioma patients. We found that if CD44 expression was to be found weak and patients were treated with surgery only then the incidence of death was significantly low as compared to patients treated with surgery followed by adjuvant therapy. A similar difference we observed for PFS, however, the data failed to reach statistical significance. Thus, CD44 might be helpful to prevent patients from unnecessary overtreatment and thereby prevent them from drug-induced toxicity. Using ROC curves, our results of CD44 as significant potential parameter for meningioma patients were confirmed. We found that CD44 could be a potential marker and it has efficacy in discriminating high and low-risk patients that might have reduced recurrence and shorter OS. However, the limitation of the present study was the small size of the patient cohort of grade 2/3 tumors compared to grade 1 tumors. Grade 2/3 tumors of meningioma are more aggressive in nature than grade 1 tumors. Therefore, further studies with a larger sample size of grade 2/3 tumors are warranted to validate our current study data.

## Conclusion

Our overall findings addressed that with CD44 immunoreactivity status meningioma patients would benefit from unnecessary overtreatment and thereby drug-induced toxicity. Also, our results revealed that CD44 could be one of the potential biomarkers that differentiate high and low-risk meningioma patients for better treatment management.

## Data Availability

The datasets generated and/or analyzed during the current study are not publicly available due to [reason why data are not public] but are available from the corresponding author on reasonable request.

## Abbreviations

ABC: Avidin-Biotin Complex
APES: 3-Amino propyl triethoxy silane
AUC: Area under curve
CD44: Cluster of differentiation 44
CI: Confidence interval
CNS: Central nervous system
DAB: 3 3′ Diaminobenzidine
DPX: Dibutylphthalate polystyrene xylene
EMT: Epithelial-mesenchymal transition
FFPE: Formalin-fixed paraffin-embedded
IHC: Immunohistochemistry
HCAM: Homing cell adhesion molecule
HR: Hazard ratio
PFS: Progression-free survival
OS: Overall survival
ROC: Receiver operating characteristic
SPSS: Statistical Package for the Social Sciences
TBS: Tris buffer saline
WHO: World Health Organization
